# Monitoring Biofouling Potential Using ATP-Based Bacterial Growth Potential in SWRO Pre-Treatment of a Full-Scale Plant

**DOI:** 10.3390/membranes10110360

**Published:** 2020-11-21

**Authors:** Almotasembellah Abushaban, Sergio G. Salinas-Rodriguez, Moses Kapala, Delia Pastorelli, Jan C. Schippers, Subhanjan Mondal, Said Goueli, Maria D. Kennedy

**Affiliations:** 1 Civil Engineering and Geosciences, Water Management Department, Delft University of Technology, Stevinweg 1, 2628 CN Delft, The Netherlands; m.kennedy@un-ihe.org; 2 IHE Delft Institute for Water Education, Environmental Engineering and Water Technology Department, Westvest 7, 2611 AX Delft, The Netherlands; s.salinas@un-ihe.org (S.G.S.-R.); mka002@un-ihe.org (M.K.); jancschippers@gmail.com (J.C.S.); 3 SUEZ International, 183 ave du 18 juin 1940, 92500 Rueil-malmaison, France; delia.pastorelli@suez.com; 4 Promega Corporation, 2800 Woods Hollow Road, Madison, WI 53711, USA; Subhanjan.Mondal@promega.com (S.M.); said.goueli@promega.com (S.G.)

**Keywords:** desalination, seawater reverse osmosis, biofouling, pre-treatment, bacterial growth potential

## Abstract

Several potential growth methods have been developed to monitor biological/organic fouling potential in seawater reverse osmosis (SWRO), but to date the correlation between these methods and biofouling of SWRO has not been demonstrated. In this research, the relation between a new adenosine triphosphate (ATP)-based bacterial growth potential (BGP) test of SWRO feed water and SWRO membrane performance is investigated. For this purpose, the pre-treatment of a full-scale SWRO plant including dissolved air flotation (DAF) and two stage dual media filtration (DMF) was monitored for 5 months using BGP, orthophosphate, organic fractions by liquid chromatography coupled with organic carbon detection (LC-OCD), silt density index (SDI), and modified fouling index (MFI). Results showed that particulate fouling potential was well controlled through the SWRO pre-treatment as the measured SDI and MFI in the SWRO feed water were below the recommended values. DAF in combination with coagulation (1–5 mg-Fe^3+^/L) consistently achieved 70% removal of orthophosphate, 50% removal of BGP, 25% removal of biopolymers, and 10% removal of humic substances. Higher BGP (100–950 µg-C/L) in the SWRO feed water corresponded to a higher normalized pressure drop in the SWRO, suggesting the applicability of using BGP as a biofouling indicator in SWRO systems. However, to validate this conclusion, more SWRO plants with different pre-treatment systems need to be monitored for longer periods of time.

## 1. Introduction

Membrane fouling is the main challenge in the operation of seawater reverse osmosis (SWRO) systems [1,2]. Pre-treatment is commonly applied to improve water quality prior to reverse osmosis (RO), and thus to minimize/mitigate the fouling issue in SWRO systems [3,4]. Almost all SWRO desalination plants require pre-treatment and the type of pre-treatment depends on the fouling potential of the raw seawater. Particulate fouling potential is commonly monitored by measuring the silt density index (SDI) and modified fouling index (MFI). Both SDI and MFI_-0.45_ are American Society for Testing and Material (ASTM) methods [5,6], in which MFI takes into account the occurrence of cake filtration [7]. It has been reported that the maximum SDI15 (SDI of 15 min) value for acceptable SWRO feed water is 3%/min [8]. 

However, to date, no standard method is available to monitor biological and organic fouling potential in SWRO systems. Monitoring biological and organic fouling potential through SWRO pre-treatment is important to improve SWRO performance [9]. For this reason, several methods have been developed and tested in SWRO desalination plants such as assimilable organic carbon (AOC) [10,11], bacterial regrowth potential (BRP) [12], membrane biofilm formation rate (mBFR) [13] and bacterial growth potential (BGP) [14]. 

The correlation between AOC and other biological/ organic/particulate fouling potential methods has been studied. Jeong and Vigneswaran [15] found excellent correlations between AOC concentration and low molecular weight neutral (LMW-N) organics concentration (R^2^ = 0.98), and between AOC and the standard blocking index calculated from MFI-UF_10 kDa_ (R^2^ = 0.97). They suggested that MFI-UF_10 kDa_ can be used as a preliminary indicator of AOC and LMW-N. Weinrich et al. [16] observed that AOC concentration neither correlated with total organic carbon (TOC) nor UV_254_ in three full-scale SWRO desalination plants. However, none of these studies attempted to correlate the AOC of RO feed water to the real time biofouling developed in the SWRO system.

Investigating the correlation between biological/organic fouling indicators in SWRO feed water and real time biofouling development in SWRO membrane systems is complicated by a few factors [14]. Firstly, using the development of head loss across the first stage of a full-scale SWRO to monitor membrane performance is complicated by the fact that several types of fouling (particulate fouling and scaling) may occur simultaneously in SWRO membrane systems. Secondly, the use of intermittent non-oxidizing biocides to combat biofouling in full-scale SWRO membrane makes establishing a real correlation between biological/organic fouling indicators in SWRO feed water and membrane performance difficult. Thirdly, cleaning in place (CIP) may be performed for other reasons than biofouling. Fourthly, to establish a real correlation, many SWRO desalination plants with different pre-treatment processes in different parts of the world need to be monitored for long periods of time with different operating conditions. Regardless of these limitations, several attempts have been made to establish the relationship between biological/organic fouling indicators and membrane performance. Hijnen et al. [17] found that the pressure drop of a membrane fouling simulator fed with fresh water depended on the AOC concentration present in the RO feed water, in which 1 µg-C/L (as acetate) added to the feed water of a membrane fouling simulator unit led to significant pressure drop in the RO membrane within 3 months. Weinrich et al. [16] reported an increase of differential pressure (0.28–0.56 bar) within 4 months with 50 µg-C/L AOC concentration in the feed water of a pilot SWRO plant. Kurihara and Ito [18] studied the relationship between mBFR and the chemical cleaning interval in 6 SWRO plants and showed that once or twice per year chemical cleaning is needed when mBFR value is less than 10. Abushaban et al. [14] monitored BGP along the pre-treatment of three full-scale SWRO desalination plants and reported a preliminary correlation between BGP in SWRO feed water and the chemical cleaning frequency in the SWRO systems.

Two main pre-treatment processes have been used to protect SWRO membranes from fouling; (i) conventional pre-treatment involving coagulation, flocculation, and particle separation, and (ii) membrane filtration systems including microfiltration and ultrafiltration [19]. The particle separation processes can consist of direct filtration with granular media, sedimentation and granular media filtration, and dissolved air flotation (DAF) and granular media filtration [20]. 

Media filtration has been widely used as a pre-treatment for SWRO systems either with or without inline coagulation. High removal of particulate, biological, and organic fouling potential has been reported by media filtration. Bonnelye et al. [21] studied the removal of SDI in a pilot SWRO plant in the Gulf of Oman (open intake) and reported that a single stage of dual media filtration (DMF) combined with 1 mg-Fe^3+^/L decreased SDI from 15 to less than 3.3%/min. Abushaban et al. [22] measured microbial adenosine triphosphate (ATP) along the pre-treatment of a full-scale SWRO desalination plant and reported more than 95% removal in DMF combined with 1.3 mg-Fe^3+^/L. Abushaban et al. [14] monitored BGP along the pre-treatment of three full-scale desalination plant, and found the highest removal (>50%) of BGP in DMF in combination with 0.8–3.6 mg-Fe^3+^/L. Similarly, Weinrich et al. [16] reported low AOC concentration (1–150 µg-C/L) in the effluent of a media filter, which later increased in RO feed water due to chemical addition [8]. 

The DAF process has been coupled with granular media filtration processes in a number of SWRO desalination plants [23]. Kim et al. [24] suggested not to use DAF alone as pre-treatment for SWRO due to limited particle removal, but rather DAF needs to be coupled with DMF to improve the pre-treatment performance. Kim et al. [24] also found that the combination of DAF with DMF further reduced the particulate fouling potential, in which SDI_-15_ and turbidity were 5.7%/min and 0.25 nephelometric turbidity units (NTU) in the filtrate of DMF (without DAF) and decreased to 4.7%/min and 0.17 NTU when DAF was coupled with DMF. However, insignificant organic matter removal was observed when DAF was coupled with DMF. Simon et al. [25] studied the removal of organics in a DAF-DMF pilot plant (coagulant dosage added into DAF system is not mentioned) located at El Prat de Llobregat (Barcelona, Spain) and reported low removal (12% of dissolved organic carbon (DOC), 33% of biopolymers, 0% humic substances, 3% of building blocks, and 10% of low molecular weight acid (LMW-A)). Moreover, Abushaban et al. [14] also reported low reduction of BGP (15%) in a DAF system (using 0.5 mg-Fe^3+^/L) in a full-scale SWRO desalination plant located in the Middle East. Shutova et al. [26] optimized the removal of organics in a seawater DAF system and reported optimum dosage of coagulant between 0.2 mg-Fe^3+^/L (at pH 5.5) and 3.5 mg-Fe^3+^/L (at pH 7.5).

Petry et al. [27] studied the effectiveness of DAF coupled with coagulation (coagulant dosage is not mentioned) prior to two-stages of DMF (El Coloso SWRO plant in Antogofasta, Chile) and reported low SDI_-15_ values (<3%/min) in SWRO feed even when frequent algal bloom events occurred in the raw seawater. In another study, Foujour et al. [28] reported SDI_-15_ values between 2% and 4%/min in SWRO feed water at the Fujairah (II) SWRO desalination plant, in which DAF is coupled with 5–6.5 mg-Fe^3+^/L coagulation/flocculation and gravity DMF. However, little data is available on the removal of biological/organic fouling potential in SWRO pre-treatment, particularly in full-scale SWRO desalination plants. 

This research aims to investigate the relationship between the BGP of SWRO feed water and the pressure drop increase and permeability decline in the SWRO system. For this purpose, biological/organic as well as particulate fouling indicators are used to monitor the pre-treatment of a full-scale SWRO desalination plant including DAF coupled with inline coagulation (1.0–1.6 mg-Fe^3+^/L) and two stages of pressurized DMF. The SWRO plant was monitored for five months in terms of turbidity, microbial ATP, particulate fouling potential (SDI and MFI), and organic indicators (total organic carbon (TOC), liquid chromatography coupled with organic carbon detection (LC-OCD)) and biological fouling potential (BGP and orthophosphate). This work also presents information on the removal of biological/organic fouling potential through the pre-treatment of SWRO, in particularly in DAF-DMF seawater systems.

## 2. Materials and Methods 

### 2.1. Description of SWRO Plant

**Figure 1:**
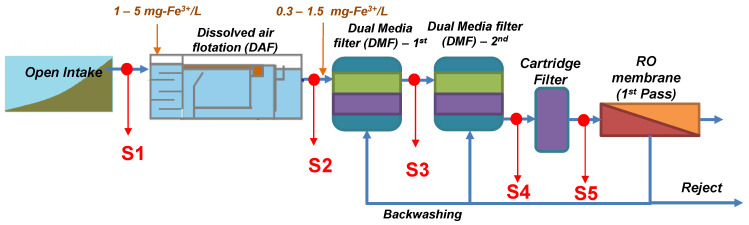
Schematic of the seawater reverse osmosis (SWRO) desalination plant with added coagulant dosage during the tested period (July–December).

The study was performed at a full-scale SWRO desalination plant fed via an open intake with seawater from the Gulf. Figure 1 shows the treatment scheme of the plant which consists of DAF combined with inline coagulation (1–5 mg-Fe^3+^/L, depending on the SDI of the raw seawater), inline coagulation (0.3–1.5 mg-Fe^3+^/L), two stage DMF, cartridge filtration (CF) with 5 µm pore size, and RO membranes. Phosphonate-based antiscalant dosed after CF. The properties of the DMFs are presented in Table 1. DMFs are backwashed using SWRO brine (from the first pass).

### 2.2. Sample Collection, Measurement, and Transportation

Seawater samples were collected every two weeks (July to December) from the main header of the seawater intake (S1), after DAF (S2), after the first stage of dual media filtration (DMF1, S3), after second stage of dual media filtration (DMF2, S4), and after CF (S5). The properties of all collected seawater samples from intake and potable water are listed in Table 2. The following indicators were measured for 5 months; turbidity, total iron, microbial ATP, particulate fouling potential indicators (SDI_-15_ and MFI_-0.45_), biological fouling potential (BGP and orthophosphate concentration) and organic indicators (such as TOC and LC-OCD).

### 2.3. Water Quality Characteristics

#### 2.3.1. SDI and MFI

The standard methods in the American Society for Testing and Material (ASTM) to measure particulate fouling potential in an RO system were used (namely; SDI [5] and MFI_-0.45_ [6]). SDI is the rate of plugging of a membrane filter having 0.45 µm pores at a pressure of 210 kPa (30 psi) for a certain period of time. Typically, SDI of 15 min (SDI_-15_) is used. It should be noted that the reported value should not exceed 75% of the maximum value (5%/min) [29]. In case of high particulate fouling potential, shorter time needs to be used such as 10 min (SDI_-10_) or 5 min (SDI_-5_). If the reported value exceeds 75% of SDI_-15_ (15%/min), then MFI_-0.45_ should be used [29]. For this study, SDI_-5_ was measured in seawater intake and SDI_-15_ was measured along the pre-treatment (after DMF1, DMF2 and CF). SDI and MFI_-0.45_ were measured using the portable SDI /MFI Analyzer (Convergence, Enschede, The Netherlands). 

#### 2.3.2. Microbial ATP 

The ATP filtration method was used to measure microbial ATP along the pre-treatment of the SWRO plant, which is described in Abushaban et al. [22]. In short, (i) seawater samples were filtered through sterile 0.1 µm PVDF membrane filters. (ii) The retained microorganisms on the membrane filter surface were rinsed with 2 mL of sterilized artificial seawater water. (iii) 5 mL of Water-Glo lysis reagent (Promega Corp., Madison, WI, USA) was filtered through the filter to extract the microbial ATP from the retained cells. (iv) ATP of the filtrate was measured by mixing 100 µL aliquot with 100 µL of ATP Water-Glo detection reagent. The average emitted light measured by the Luminometer (GloMax®-20/20, Promega Corp., Madison, WI, USA) was converted to microbial ATP concentration based on a calibration curve. Microbial ATP was measured on site. For each sample, six replications were measured. 

#### 2.3.3. Bacterial Growth Potential (BGP)

Seawater samples were pasteurized (70 °C for 30 min) on-site to inactivate marine microorganisms and shipped to IHE Delft facilities (Delft, The Netherlands) for analysis. All samples were collected in AOC-free 100 mL Duran® laboratory glass bottles with tight-fitting screw caps and transported in a cooler at 5 °C within 36 h. BGP was measured following the described method by Abushaban et al. [30]. In short, the pasteurized sample was distributed in triplicate in 30 mL carbon-free vials and each vial was inoculated with 10,000 cells/mL (intact cell concentration measured by flow cytometry) of an indigenous microbial consortium. Samples were incubated at 30 °C and bacterial growth was monitored using microbial ATP measurement in seawater for 5 days. BGP was calculated based on a calibration line between for carbon and BGP at constant temperature [22]. 

#### 2.3.4. Liquid Chromatography—Organic Carbon Detection (LC-OCD) 

LC-OCD was used to measure the chromatography dissolved organic carbon (CDOC) and organic fractions including biopolymers, humic substances and low molecular weight (LMW) acids. The LC-OCD system separates dissolved organic carbon (DOC) compounds using a size exclusion chromatography column, followed by multi detection of organic carbon, UV-absorbance at 254 nm (UV_254_) and nitrogen determination (DOC-Labor, Karlsruhe, Germany). Seawater samples were measured monthly according to the protocol described by Huber et al. [31]. Seawater samples were shipped in a cooler box (5 °C) to DOC-Labor Huber lab (Karlsruhe, Germany) for analysis.

#### 2.3.5. Total Organic Carbon (TOC) 

TOC concentration in seawater was measured using a Shimadzu TOC-VCPN (Kyoto, Japan) analyzer based on combustion catalytic oxidation/nondispersive infrared sensor (NDIR) method. The sample was measured in duplicate and without pre-treatment (filtration). Thus, the measured TOC concentration includes both dissolved and particulate carbon. The limit of detection of TOC measurement is 0.2 mg/L. 

#### 2.3.6. Orthophosphate Concentration

Orthophosphate analysis was performed using Skalar San^++^ analyzer (Skalar, Breda, The Netherlands) at the facility of Rijkswaterstaat (Lelystad, The Netherlands). Molybdate reagent and ascorbic acid were added to the seawater samples at a temperature of 37 °C. The added molybdate and the orthophosphate present in seawater samples form a phosphor-molybdate complex in the acidic environment after reduction with ascorbic acid and in the presence of antimony. This gave a blue colored complex, which was measured at 880 nm using a 50 mm cuvette and a spectrophotometer. The limit of detection of the orthophosphate analysis is 0.3 µg/L.

## 3. Results

### 3.1. Turbidity 

Turbidity after the intake ranged between 0.5 and 2.9 NTU (Table 3). The highest turbidity (~2.9 NTU) in the seawater was measured in August, which was also confirmed by the SDI_-15_. The measured turbidity after DMF2 and CF were very low (<0.1 NTU), indicating that most of the colloidal particles were removed through the two stages of media filtration. The removal of turbidity in DMF is also consistent with the reported values in the literature [24,32,33]. Overall, more than 90% of turbidity was removed during the pre-treatment of SWRO (from S1 to S5). 

### 3.2. Particulate Fouling Indices

#### 3.2.1. Silt Density Index (SDI)

High SDI values were measured (Table 3) in the seawater intake during the summer (July and August), which are above the maximum limit (SDI_-5_ = 15%/min) defined by ASTM [29]. The measured SDI_-15_ after DMF1 ranged between 3.5 and 5.2%/min (with an average of 4.4%/min) and further decrease after passing through DMF2 to 3.3%/min. The measured SDI_-15_ after DMF is close to the reported values by Bonnelye et al. [21], who reported SDI_-15_ below 3.3%/min after DMF. Some of the literature has reported even higher SDI_-15_ (>6.6%/min) after DMF [32]. As expected, negligible improvement in SDI_-15_ was observed through the CF. Overall, the measured SDI_-15_ after CF was below the recommended SDI_-15_ values (<4%/min) by the membrane manufacturers, indicating low particulate fouling potential in the SWRO feed water.

#### 3.2.2. Modified Fouling Index (MFI_-0.45_)

High MFI_-0.45_ variations were observed in the seawater intake, ranging between 22 to 60 s/L^2^ (Table 3). The measured MFI_-0.45_ values are lower than the reported values by Salinas Rodriguez et al. [34] in the raw seawater of the North Sea (20–250 s/L^2^), suggesting lower particulate fouling potential in the monitored SWRO plant. Similar to SDI, significant removal of MFI_-0.45_ was observed in DMF1 and DMF2, in which MFI_-0.45_ decreased from 41 s/L^2^ in the seawater intake to 3.4 s/L^2^ after DMF1 and to 1.7 s/L^2^ after DMF2. Shrestha et al. [33] reported slightly higher MFI_-0.45_ values after lab-scale sand filters (1.9–5.9) even though the MFI_-0.45_ of influent was much lower (4–10 s/L^2^). Slight improvement of MFI_- 0.45_ was found after the cartridge filter, in which the average measured MFI_-0.45_ in the SWRO feed water was 1.3 s/L^2^. Overall, 97% removal of MFI_-0.45_ was achieved in the SWRO pre-treatment.

### 3.3. Biomass Quantification

**Figure 2:**
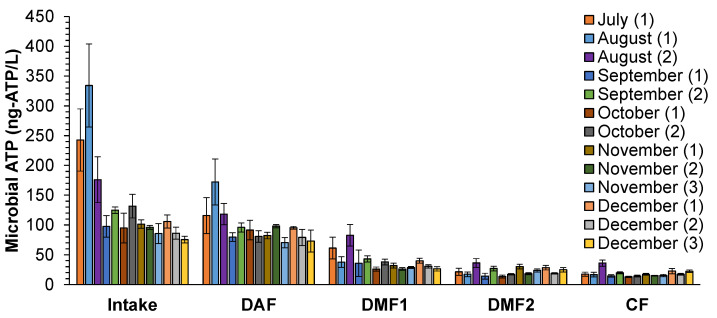
Microbial ATP concentrations in the SWRO pre-treatment train over a 5 month period (n = 13). DAF (dissolved air flotation), DMF1 (the first stage of dual media filtration), DMF2 (the second stage of dual media filtration) and CF (cartridge filtration). The number mentioned in the legend refers to the batch of samples collected in the month.

Microbial ATP concentration in the seawater intake varied from 75 to 335 ng-ATP/L (Figure 2). High microbial ATP concentrations (>>100 ng-ATP/L) were observed in July and August, which could be attributed to microbial growth as a result of high temperature of the water (32–40 °C) in July and August, whereas, from September to December, microbial ATP concentrations fluctuated around 100 ng-ATP/L. This was also observed in the North Sea water by Abushaban et al. [30] who reported high seasonal variations in microbial ATP concentrations ranging between 25 and 1000 ng-ATP/L.

Monitoring microbial ATP through the pre-treatment showed that, on average, 27% of microbial ATP was removed through the DAF system, in which microbial ATP concentrations after DAF ranged between 50 and 170 ng-ATP/L. Significant removal of microbial ATP (60%) was found in DMF1 in combination with inline coagulation (0.3–1.5 mg-Fe^3+^/L). This is close to the reported removal (65–85%) in a pilot seawater media filter (without coagulation) fed with seawater from the North Sea [22]. Further removal of microbial ATP was seen in the DMF2 (45%) and the CF (16%). Microbial ATP concentration in the SWRO feed water ranged between 10 and 35 ng-ATP/L. In total, more than 86% of microbial ATP was removed through the SWRO pre-treatment. Abushaban et al. [22] reported higher removal of microbial ATP (95%) in a full-scale SWRO plant with two stages of DMFs (with similar properties as the DMFs of the monitored plant) coupled with inline coagulation (1.3 mg- Fe^3+^/L). The higher removal of microbial ATP is attributed to the higher coagulant dosage prior to DMF in this study.

### 3.4. Organic Matters

#### 3.4.1. Total Organic Carbon

High TOC concentration was measured in the seawater intake ranging between 1.9 and 4.4 mg/L (Table 4) with an average of 2.9 mg/L. After DAF and DMF1, the TOC concentration declined to 2.3 mg/L (15%) and 2.0 mg/L (13%), respectively. The removal is close to that reported in the literature. Shutova et al. [26] reported 16% removal of DOC in a lab scale DAF system fed with Gold Coast seawater and with 1 mg-Fe^3+^/L. Jeong et al. [9] observed 0.1 mg/L (12%) removal of DOC in the DMF of Perth SWRO desalination plant. Slight TOC removal was found through DMF2 (5%) and after CF (6%). 

The overall removal of TOC along the pre-treatment is 33%. However, even lower removal of TOC is reported in the literature. Weinrich et al. [35] reported only 3–6% removal of TOC along the pre-treatment (coagulation (dosage is not reported), sand filter, diatomaceous filter and cartridge filter) of the Tampa Bay seawater desalination plant (FL, USA) and no removal of TOC through the pre-treatment (ultrafiltration and cartridge filter) of a pilot plant in Moss Landing (CA, USA) fed with seawater from Monterey Bay. Moreover, Poussade et al. [36] found that TOC decreased from 1.14 to 0.89 mg/L (13.5%) through the pre-treatment (coagulation with 1 mg-Fe^3+^/L, flocculation and sand filtration) of a SWRO pilot plant fed with seawater from the Gulf of Oman. This lower removal percentage of TOC is because TOC concentration may include a high percentage of non-biodegradable organic carbon, and that the applied coagulant dosage was low.

#### 3.4.2. Organic Fraction by LC-OCD Analysis

TOC concentrations along the pre-treatment were higher (35% in average) than the measured hydrophilic dissolved organic carbon (CDOC) concentrations which could be due to particulate carbon and/or the higher sampling frequency of TOC (biweekly) comparing to CDOC (monthly). In total, 384 µg-C/L of CDOC was removed (21%) through the SWRO pre-treatment (Table 4). The highest removal of CDOC was measured in the DAF and DMF1 (7% and 9%, respectively) which is rather limited. This low removal of CDOC is in agreement with the reported removal by Simon et al. [25]. The CDOC removal in DAF (135 µg-C/L) was mainly due to the removal of humic substances (78 µg-C/L) and biopolymers (67 µg-C/L). Similar findings were reported at a bench-scale DAF system by Shutova et al. [26]. The high removal of humic substances in the DAF was also confirmed by the monitored fluorescence excitation emission matrix (FEEM) (See Figure S1).

Slightly higher removal of biopolymers, humic substances and low molecular weight acid (LMW acids) were observed in DMF2 (38, 16 and 5 µg-C/L, respectively) than DMF1 (21, 8, and 9 µg-C/L, respectively), probably due to smaller media size in DMF2 (Table 1). The low removal of humic substances in DMF2 was expected as humic substances are mainly removed by coagulation. Shrestha et al. [33] reported only 2% removal of humic substances in sand and anthracite biofilters. CF showed no removal of organic carbon, as expected. Overall, low removal of organic fractions was seen through the pre-treatment of the SWRO desalination plant, with the best removal in the DAF system.

### 3.5. Biofouling Indicators

#### 3.5.1. Orthophosphate

The orthophosphate concentration measured in the seawater intake ranged between 2 and 11 µg-PO_4_-P/L (Table 5). Munshi et al. [37] measured orthophosphate concentration in the raw seawater (Arabian Gulf) and the permeate of nano-filtration of Al-Jubail SWRO desalination plant and reported 4.7 and 1.1 µg-PO_4_-P/L, respectively. Significant removal (68%) of orthophosphate was observed through DAF and further removal (33%) was found through DMF1. The high removal of phosphate in the DAF and DMF1 could be attributed to the precipitation of iron phosphate as coagulant 1-5 mg-Fe^3+^/L was added prior to DAF and DMF1 [38]. It is worth mentioning that no data is available in the literature on the removal of orthophosphate in the pre-treatment processes of SWRO membrane systems. Similar to BGP and TOC, orthophosphate concentration increased after CF from 1.1 to 1.5 µg-PO_4_-P /L, which may be attributed to the addition of phosphonate antiscalant and/or to the presence of nutrients in the make-up water.

#### 3.5.2. Bacterial Growth Potential

**Figure 3:**
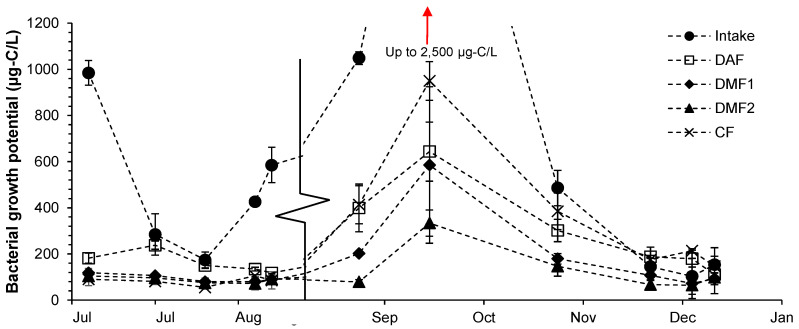
BGP along the pre-treatment of the SWRO desalination plant.

High BGP variations were observed in the seawater intake, in which BGP ranged between 200 and 2500 µg-C/L as glucose (Figure 3). Extremely high BGPs were observed from the end of August to October in the seawater intake and along the pre-treatment due to algal blooms. Algal blooms in the Arabian Sea in September and October are widely reported [39,40]. It is believed that higher BGP in the summer might be attributed to carbon release from the algal cells present in seawater. 

The highest BGP removal was found through DAF (52%) and DMF1 (40%). This result is in agreement with the findings of Kim et al. [24] who reported similar removal of organic fractions in terms of chemical oxygen demand (35%), UV_254_ (23%) and chlorophyll-a (45%) in both DAF and DMF when combined with inline coagulation (1.3 mg-Fe^3+^/L) The high removal of BGP in DAF could be attributed to the coagulant dosage within DAF (1–1.6 mg-Fe^3+^/L), while the achieved removal of BGP in DMF1 may be due to the applied inline coagulant dosage (0.35 mg-Fe^3+^/L) prior to DMF1 and/or due to biodegradation in DMF1. Abushaban et al. (2019) reported slightly higher BGP removal where 44% in a pressurized pilot media filter without coagulation dosage and 55% in a gravity DMF combined with inline coagulation (3.6 mg-Fe^3+^/L) were obtained [22,30].

Slight removal (14%) of BGP was also noted through DMF2, which may be due to the shorter contact time compared with DMF1 and/or the absence of coagulant dosage. One may expect higher organic biodegradation in DMF2 because the filtration cycle of DMF2 is longer (>40 h) compared to DMF1 (~24 h). The long filtration time may allow the development of a substantial biofilm on the filter media. However, the use of SWRO brine to backwash DMF may have hindered the initial formation of biofilm due to the osmotic shock expressed by the bacteria. This has been verified by monitoring microbial ATP in the filtrate of the DMFs (See Figure S2). These results could suggest an impact of using SWRO brine to backwash media filters on biofilm development.

Higher BGP was observed after the CF which could be attributed to the addition of antiscalant [41] or the make-up water used for diluting antiscalant (See Table S1). Higher organic concentration after antiscalant addition has been observed in several SWRO and RO plants [9,42]. On average, BGP was reduced from 373 µg-C/L (as glucose equivalent) in the seawater intake to 146 µg-C/L (as glucose equivalent) in the SWRO feed water. The removal of BGP (62%) along the SWRO pre-treatment is comparable to the reported BGP removal of 50–72% by Abushaban et al. [14] in three full-scale SWRO desalination plants with different pre-treatment processes. 

## 4. Discussion

Several parameters have been monitored along the SWRO pre-treatment and in the SWRO feed water including particulate, biological and organic fouling indicators over a 5 months period. It is assumed that scaling did not occur as antiscalant is dosed prior to the SWRO membranes and thus should eliminate the occurrence of any scale in the first pass of the SWRO plant. 

### 4.1. Turbidity 

Significant removal of turbidity was observed through the pre-treatment. The measured turbidity (<0.1 NTU) in the SWRO feed were below the recommended values (<0.1 NTU) according to the membrane manufacturer.

### 4.2. Particulate Fouling

Results, in terms of SDI and MFI_-0.45_, showed that particulate fouling was well controlled through the pre-treatment. This can be justified by several observations. Firstly, high removal (>80%) of particulate fouling indices (SDI_-15_ and MFI_-0.45_) was observed through the SWRO pre-treatment, in which the highest removal was achieved in DMF1 combined with 1–4.5 mg-FeCl_3_/L as an inline coagulation. The high removal in the DMF is in agreement with what was reported earlier [34,43]. Secondly, the measured SDI_-15_ (3.2 ± 0.7) in the SWRO feed was below the manufacturer’s recommended values (<4%/min). Thirdly, applying the particulate fouling prediction model (based on MFI) presented by Salinas Rodriguez et al. [34], the SWRO system can be operated for more than two years before observing a one bar increase in the net driving pressure of the SWRO membrane system for MFI < 2 s/L^2^ in the SWRO feed water. Nevertheless, it is possible that particles smaller than 0.45 µm may pass through pre-treatment and contribute to fouling development. Therefore, we recommend, in addition to SDI and MFI_-0.45_, measuring also the MFI-UF values of RO feed water to completely rule out the contribution of colloidal particles to fouling.

The measured turbidity values (<0.1 NTU) were quite low; however, it has been reported that turbidity does not correlate with particulate fouling potential [21].

### 4.3. Biomass Quantification 

Having high microbial concentration in the SWRO feed water does not directly cause biofouling. It may cause particulate fouling and/or accelerate bacterial growth in the SWRO membrane system and thus indirectly may increase the rate of biofouling. Significant removal of microbial ATP (85%) was also observed through the SWRO pre-treatment (Figure 2), in which microbial ATP concentration decreased, on average, from 130 ng-ATP/L in the raw seawater intake to 18 ng-ATP/L in the SWRO feed water. The microbial ATP concentration in the SWRO feed water is equivalent to 20,000 intact cells per mL (using the reported correlation between microbial ATP and intact cell concentration of North Sea water [22]). 

### 4.4. Biological and Organic Fouling Potential in the Pre-Treatment

Compared to the removal of particulate fouling potential and microbial ATP, lower removal percentages of biological/organic fouling potential were seen along the SWRO pre-treatment train. However, DAF combined with 1–5 mg-Fe^3+^/L coagulant dosage showed reasonable removal of biological/organic fouling potential, in which 3.6 µg-PO_4_-P/L of orthophosphate (68%), 197 µg-C/L of BGP (52%), 77 µg-C/L of biopolymers (25%), 135 µg-C/L of CDOC (7%), and 77 µg-C/L of humic substances (10%) were removed. Shutova et al. [26] studied the removal of organic matter in the DAF system, used as pre-treatment for SWRO membrane. The magnitude of removed organic fractions (biopolymers: 60–65 µg-C/L, CDOC: 140–240 µg-C/L and humic substances: 100–180 µg-C/L) are in the same range as the observed removal in this study. 

Good removal of biological/organic fouling potential was measured in DMF1 combined with 1–5 mg-FeCl_3_/L of coagulation compared to the reported removal in the literature. The observed removal of BGP (74 µg-C/L, 40%), CDOC (143 µg-C/L, 9%), biopolymers (21 µg-C/L, 10%) and humic substances (9 µg-C/L, 1.3%) in DMF1 were higher than reported by Jeong et al. [9] in the DMF of Perth SWRO desalination plant, in which they reported 13% of AOC (5 µg-C/L), 6.6% of CDOC (100 µg-C/L), 11% of biopolymers (10 µg-C/L), and 0% of humic substances. Moreover, the overall removal of organic fractions in the DAF and the DMF1 of the studied plant is higher than the reported organic removal by Simon et al. [25], after DAF (coagulant dosage not mentioned) and DMF, of a pilot plant located at El Prat de Llobregat (Barcelona, Spain), in which 161 µg-C/L of CDOC (12%), 35 µg-C/L of biopolymers (13%), 0 µg-C/L of humic substances (0%) and 6 µg-C/L of LMW-acid (10%) were removed. 

These results reveal that the achieved removal of biological/organic fouling potential in the monitored SWRO plant is comparable to SWRO plants at different locations, and even higher than some SWRO plants. However, even better removal of biological/organic fouling potential could be achieved by adjusting several design and operational parameters. For instance, extending the contact time of the DMF is expected to enhance biodegradation of organics. Moreover, the use of SWRO brine to backwash the media filters could burst the microorganisms/biofilm in the media filtration and thus affect the biodegradation rate in DMF, because the high osmotic pressure that the biofilm is exposed to (during backwashing).

### 4.5. Biological/Organic Fouling Potential in the SWRO Feed

Although reasonable concentration of organic and biological fouling potential was removed through the pre-treatment, still considerable concentration remains in the SWRO feed water (Table 4 and Table 5). As no standard threshold value for organic and biological fouling potential is available, the measured concentration in the SWRO feed water is firstly compared with those reported in the literature. According to the literature, the fouling in the SWRO system is most likely due to biofouling for the following reasons; (i) Jeong et al. [9] observed biofouling in the SWRO system at the Perth desalination plant where lower organic fractions (1.3 mg/L of CDOC, 50 µg-C/L of biopolymers, 140 µg-C/L of humic substances) in the SWRO feed water were found which are lower than those measured as organic fractions (1.4 mg/L of CDOC, 141 µg-C/L of biopolymers and 623 µg-C/L of humic substances). (ii) Weinrich et al. [16] reported a preliminary AOC threshold concentration of 50 µg-C/L based on pilot tests, while 146 µg-C/L of BGP was measured in the SWRO feed (assuming AOC and BGP are similar). Thus, it was suggested that biofouling in the SWRO membrane occurred due to high potential of biological/organic fouling in the SWRO feed water. 

### 4.6. Investigating the Relation between Membrane Performance and BGP in SWRO Feed Water

**Figure 4:**
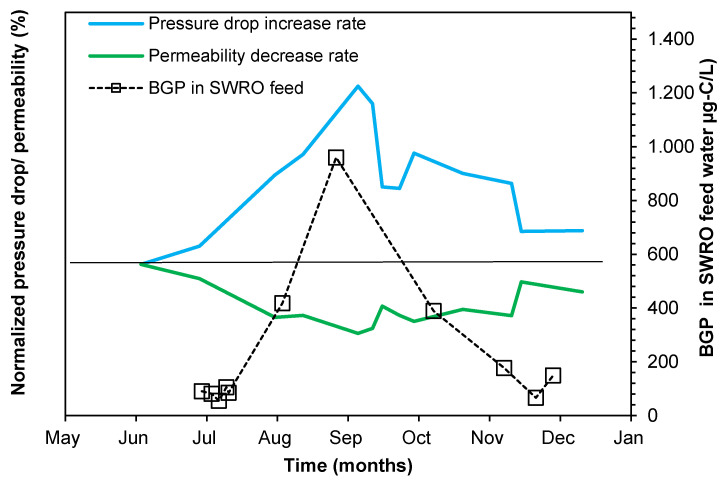
Correlation between BGP in the SWRO feed water and the normalized pressure drop and normalized permeability in the SWRO membrane system (n = 11).

The relationship between BGP in the SWRO feed water and the normalized pressure drop/permeability in the SWRO membrane system was studied (Figure 4) and it was found that higher BGP was measured from July to September, corresponding to a higher normalized pressure drop. The measured BGP in the SWRO feed water in July were all at around 100 µg-C/L, during which time the normalized pressure drop further inclined and the normalized permeability also further slowly declined, suggesting that 100 µg-C/L of BGP may still be sufficient to cause biofouling in SWRO membrane systems. This result suggests that BGP could be used to monitor biological fouling in the SWRO system. However, more data need to be generated at different SWRO plants at different locations to validate the use of BGP as a biological fouling indicator.

## 5. Conclusions

- Seasonal seawater quality variations were observed in the seawater intake in terms of silt density index (SDI), modified fouling index (MFI), microbial ATP, bacterial growth potential (BGP), orthophosphate and total organic carbon.
- Particulate fouling was well controlled by the SWRO pre-treatment, in which the measured SDI_-15_ (<3.2%/min), MFI_-0.45_ (<1.8 s/L^2^) and turbidity (<0.1 NTU) in the SWRO feed water were all below the recommended values. The highest removal (70–90%) of SDI_-15_, MFI_-0.45_ and turbidity was achieved in the first stage of dual media filtration when combined with inline coagulation (0.3–1.5 mg-Fe^3+^/L).
- Despite achieving more than 75% removal of biological/organic fouling potential along the SWRO pre-treatment, particularly in the dissolved air flotation and the first stage of dual media filtration, BGP and orthophosphate concentrations increased by 35% in the SWRO feed due to chemical addition, and/or due to nutrients present in the water storage tanks or make-up water.
- Investigating the relation between normalized pressure drop in the SWRO system and Bacterial Growth Potential in the SWRO feed water showed that the growth potential measured in the SWRO feed water from 100 to 950 µg-C/L led to an increase in the normalized pressure drop within 3 months. This result may suggest the applicability of using Bacterial Growth Potential of SWRO feed water as a biological fouling indicator in SWRO systems. However, to ensure the validity of this conclusion, more SWRO plants need to be monitored at different locations for longer periods of time.

## Figures and Tables

**Table 1:** Characteristics and operational properties of the two stage dual media filtration (DMF).

Parameter	1st Stage of DMF	2nd Stage of DMF
No. and type of filters	24 horizontal pressure filters	16 horizontal pressure filters
Surface area	51 m^2^	51 m^2^
Filtration rate	12.5 m/h	19.5 m/h
Filtering media	0.55 mm sand and 1.50 mm anthracite	0.28 mm sand and 1.2 mm anthracite
Filtration cycle duration	~24 h	>40 h

**Table 2:** The water properties of influent and potable water.

Parameter	Feed Water	Potable Water
pH	8.1–8.3	6.8–7.1
Turbidity	0.8–2.9 NTU	0.01–0.06 NTU
Total dissolved solids	45–48 g/L	≤150
Temperature	22–40 °C	22–40 °C
Boron	-	1.1–1.7 mg/L

**Table 3:** Turbidity, silt density index (SDI) and modified fouling index (MFI)_-0.45_ along the pre-treatment of the SWRO plant over a period of 5 months (n = 20).

Parameter	Statistics	Seawater Intake	After DMF1	After DMF2	SWRO Feed	Overall Removal
Turbidity (NTU)	Min.	0.4	NA	<0.1	<0.1	0.3
	Max.	2.9	NA	0.2	0.2	2.6
	Mean	1.5	NA	<0.1	<0.1	1.4 ± 0.9
SDI_-15_ (%/min)	Min.	9 *	3.5	2.8	2.6	6
	Max.	>15 *	5.2	3.9	<4	>11
	Mean	>15 *	4.4 ± 0.5	3.3 ± 0.4	3.2 ± 0.7	>11
MFI_-0.45_ (s/L^2^)	Min.	22	1.6	1.5	0.6	22
	Max.	60	4.4	2.1	1.8	59
	Mean	41 ± 20	3.4 ± 1.2	1.7 ± 0.3	1.3 ± 0.5	39.7 ± 20

* SDI_-5._

**Table 4:** Removal of total organic carbon *(TOC) (n = 12) and various fractions of organic carbon (n = 5) along the pre-treatment of SWRO desalination plant over 5 months period.

Parameter		Seawater intake	After Dissolved Air Flotation (DAF)	After Dual Media Filtration (DMF)1	After DMF2	After Cartridge Filtration (CF)	Overall Removal
Coagulation (mg-Fe^3+^/L)		-	1–5	0.3–1.5	-	-	
TOC (mg/L)	Mean (%removal)	2.9 ± 0.8	2.3 ± 0.3 (15%)	2.0 ± 0.2 (13%)	1.9 ± 0.2 (5%)	1.8 ± 0.1 (6%)	0.9 ± 0.6 (33%)
Chromatography dissolved organic carbon (CDOC ) (µg-C/L)	Min.	1543	1409	1400	1317	1236	307
	Max.	2026	1911	1589	1711	1679	573
	Mean (%removal)	1808 ± 244	1673 ± 268 (7%)	1530 ± 90 (9%)	1468 ± 174 (4%)	1424 ± 190 (3%)	384 ± 127 (21%)
Biopolymers (µg-C/L)	Min.	216	165	160	120	126	89
	Max.	339	236	196	152	149	192
	Mean (%removal)	265 ± 57	198 ± 35 (25%)	177 ± 19 (11%)	140 ± 15 (21%)	141 ± 10 (0%)	124 ± 51 (47%)
Humic substances (µg-C/L)	Min.	577	529	540	511	481	58
	Max.	881	796	764	755	755	143
	Mean (% removal)	737 ± 165	660 ± 147 (10%)	651 ± 125 (1%)	635 ± 132 (2%)	623 ± 143 (2%)	114 ± 38 (15%)
Low molecular weight (LMW)-acid (µg-C/L)	Min.	115	121	115	106	102	4
	Max.	203	192	183	181	175	35
	Mean (%removal)	157 ± 47	157 ± 37 (0%)	149 ± 36 (5%)	144 ± 39 (3%)	139 ± 38 (3%)	18 ± 13 (11%)

**Table 5:** Orthophosphate and bacterial growth potential (BGP) along the pre-treatment of the SWRO desalination plant over a 5 month period.

Parameter		Seawater Intake	After DAF	After DMF1	After DMF2	After CF & Antiscalant	Overall Removal
Coagulation (mg-Fe^3+^/L)		-	1–5	0.3–1.5	-	-	
Orthophosphate (µg-PO_4_-P/L)	Min.	1.8	1.0	0.6	0.7	1.1	
	Max.	11	2.6	1.5	1.5	2.6	
	Mean (%removal)	5.3 ± 3.7	1.7 ± 0.6 (68%)	1.1 ± 0.4 (35%)	1.1 ± 0.2 (0%)	1.5 ± 0.6 (−36%)	3.8 ± 3.6 (72%)
BGP (µg-C/L)	Min.	105	112	72	65	55	
	Max.	2500	650	590	330	950	
	Mean (%removal)	373 ± 268	180 ± 61 (52%)	106 ± 32 (40%)	92 ± 25 (14%)	146 ± 106 (−37%)	227 ± 660 (62%)

## Supplementary Materials

The following are available online at https://www.mdpi.com/2077-0375/10/11/360/s1, Figure S1: FEEM fluorescence features along the SWRO pre-treatment for seawater samples collected in August 2018, Figure S2: Hourly monitoring of microbial ATP concentrations for the seawater intake, after DAF, DMF1 and DMF2. Table S1: The effect of antiscalant addition on BGP measurement.
