# Supplementary material for: Monitoring Biofouling Potential Using ATP-Based Bacterial Growth Potential in SWRO Pre-Treatment of a Full-Scale Plant
Source: Membranes (Basel). 2020 Nov 21;10(11):360. doi: 10.3390/membranes10110360 (PMC7700145; doi:10.3390/membranes10110360)
Supplement: Supplementary file 1 [file membranes-10-00360-s001.pdf]

Supplementary Materials: Monitoring Biofouling Potential Using ATP-Based Bacterial Growth Potential in SWRO Pre-Treatment of a Full-Scale Plant

Almotasembellah Abushaban [1,2,*], Sergio G. Salinas-Rodriguez [2], Moses Kapala [2], Delia Pastorelli [3], Jan C. Schippers [2], Subhanjan Mondal [4], Said Goueli [4] and Maria D. Kennedy [1,2]

[1] Civil Engineering and Geosciences, Water Management Department, Delft University of Technology, Stevinweg 1, 2628 CN Delft, Netherlands; a.abushaban@un-ihe.org (A.A.); m.kennedy@un-ihe.org (M.D.K.).

[2] IHE Delft Institute for Water Education, Environmental Engineering and Water Technology Department, Westvest 7, 2611 AX, Delft, Netherlands; s.salinas@un-ihe.org (S.G.S.-R.); jancschippers@gmail.com (J.C.S.)

[3] SUEZ International, 183 ave du 18 juin 1940, 92500 Rueil-malmaison, France; delia.pastorelli@suez.com (D.P.)

[4] Promega Corporation, 2800 Woods Hollow Road, Madison, WI 53711, USA; Subhanjan.Mondal@promega.com (S.M.); said.goueli@promega.com (S.G.)

* Correspondence: a.abushaban@un-ihe.org or motasem.abushaban@gmail.com

Received: 30 October 2020; Accepted: 19 November 2020; Published: date

Annex A: Fluorescence excitation-emission matrix (FEEM) spectroscopy

The fluorescence emitting organic substances in seawater samples were measured using a FluoroMax-3 spectrophotometer (Horiba Jobin Yvon, Inc., Edison, New Jersey, USA) with a 150 W ozone-free xenon arc lamp for excitation. The seawater samples were scanned over the excitation wavelength range from 240 to 450 nm, and an emission wavelength range of 290 to 500 nm to produce a three-dimensional matrix. Before FEEM analysis, the dissolved organic carbon (DOC) of seawater samples was measured using Shimadzu TOC, and diluted with MilliQ water to get a DOC concentration of approximately 1 mg/L. Excitation and emission matrices were analysed using MatLab R 2011a, and the results were analysed as described by Leenheer et al. [1].

High peaks were observed above 380 nm of excitation indicating the presence of high fraction of humic- like (marine) and humic-like components (Figure S1). No clear peak of protein-like was seen (excitation range from 310–360 nm). Clear removal of humic-like compounds was observed through the pre-treatment, in particularly, through DAF and DMF1, in which the intensity decreased to half (from 2×10^{-3} to 1×10^{-3}). This is in agreement to the findings of Shutova et al. [2] who reported 46 – 49 % removal of humic-like substances in bench-scale seawater DAF system. Slight removal of humic-like component was also seen in DMF2. Similar trend was also seen for the fulvic acid- like substances but with higher intensity (more than 3.5×10^{-3}) indicating higher contribution of fulvic acid-like compounds than humic acid-like.

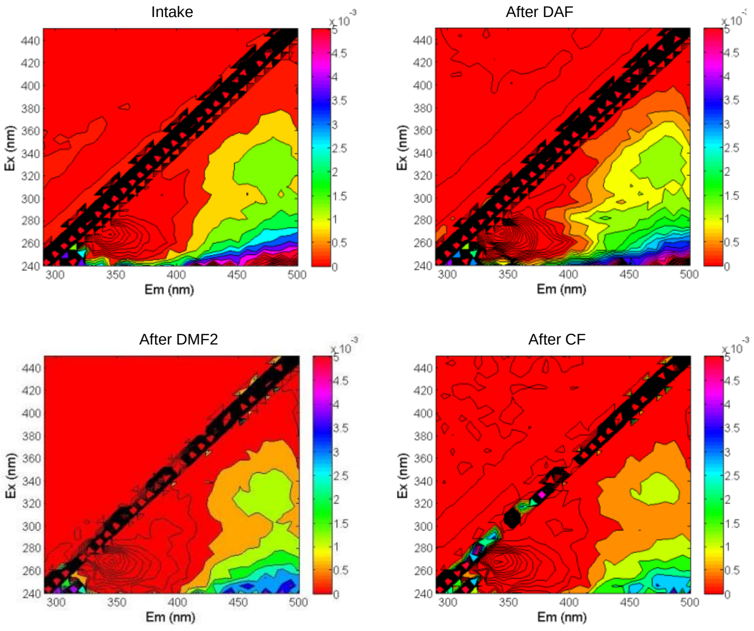

Figure S1. FEEM fluorescence features along the SWRO pre-treatment for seawater samples collected in August 2018

Annex B: Hourly Monitoring of Microbial ATP Over the Day

Microbial ATP concentration was monitored over two days along the pre-treatment and significantly variation was found in the seawater intake (between 150 and 450 ng-ATP/L) (Figure S2). Microbial ATP concentration after DAF was stable at 130 ng-ATP/L for 2 days showing the added value of DAF in stabilizing the seawater. Microbial ATP concentration ranged between 50 and 80 ng-ATP/L and between 5 and 25 ng-ATP/L before the backwashing of DMF1 and DMF2, respectively. Immediately after backwashing, low microbial ATP concentrations was observed after DMF1 and DMF2 due to the addition of high saline seawater (SWRO brine) to backwash the DMFs. High saline seawater could burst/kill the microorganisms and biofilm present in the media filters. An increase of microbial ATP concentration was observed during maturation period, which is attributed to the replacement of SWRO brine with the real seawater (less saline). The microbial ATP concentration measured after maturation was higher than the microbial ATP concentration measured before backwashing of DMF1 and DMF2 which may indicate that maturation time after backwashing could be optimized.

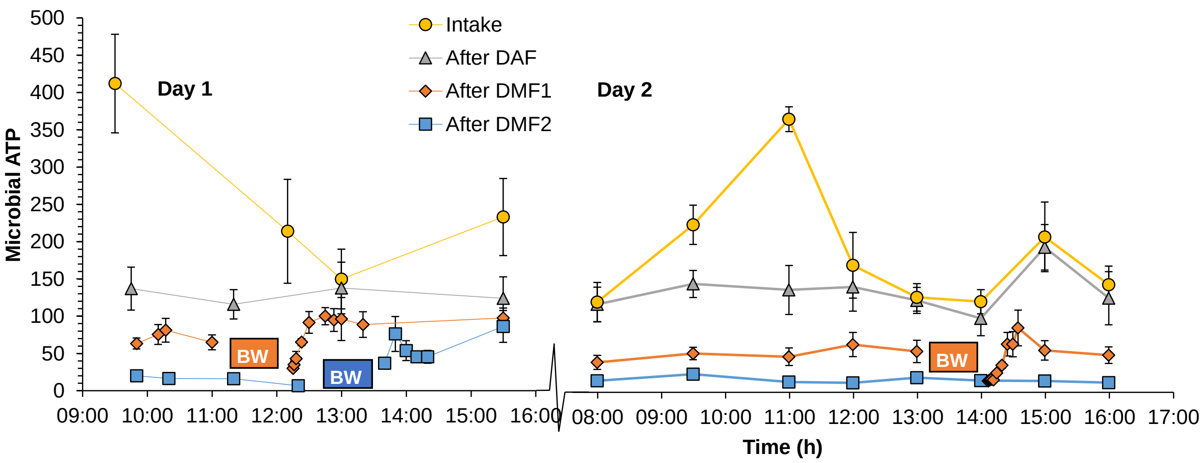

Figure S2. Hourly monitoring of microbial ATP concentrations for the seawater intake, after DAF, DMF1 and DMF2. BW = Backwashing time.

Annex C: Effect of Antiscalant Addition

The BGP, TOC and orthophosphate concentration increased after the cartridge filter, in which the highest increase was 60 % in the BGP (from 92 to 146 μg-C/L). This could probably be attributed to addition of antiscalant after the cartridge filter. Therefore, the effect of antiscalant (similar to the applied dosage in the plant) was investigated by comparing BGP of a seawater sample with and without addition of the antiscalant. It was found that the BGP increased by 28 % comparing to the sample without antiscalant as shown in Table S1. This increment percentage (28 %) is lower than the measured increment after cartridge filtration (60 %) suggesting that antiscalant partially contributed in the higher biological/organic fouling indicators after cartridge filter. The rest 32 % might be due to the presence of micro-organism in the water used for diluting the antiscalant in the full-scale SWRO desalination plant.

Table S1. The effect of antiscalant addition on BGP measurement.

	**BGP (μg/L)**
BGP without Antiscalant	150
BGP with Antiscalant	192

References

1. Leenheer, J.A.; Croué, J.-P. Peer Reviewed: Characterizing Aquatic Dissolved Organic Matter. *Environ. Sci. Technol.* **2003**, *37*, 18A–26A, doi:10.1021/es032333c.
2. Shutova, Y.; Karna, B.L.; Hambly, A.; Lau, B.; R.K. UNSW Faculty of Engineering Civil & Environmental Engineering R.K Henderson; Le-Clech, P. Enhancing organic matter removal in desalination pretreatment systems by application of dissolved air flotation. *Desalination* **2016**, *383*, 12–21, doi:10.1016/j.desal.2015.12.018.
